# Integrated transcriptomic meta-analysis and comparative artificial intelligence models in maize under biotic stress

**DOI:** 10.1038/s41598-023-42984-4

**Published:** 2023-09-23

**Authors:** Leyla Nazari, Muhammet Fatih Aslan, Kadir Sabanci, Ewa Ropelewska

**Affiliations:** 1 Crop and Horticultural Science Research Department, Fars Agricultural and Natural Resources Research and Education Center, Agricultural Research, Education and Extension Organization (AREEO), Shiraz, Iran; 2 Electrical and Electronics Engineering, Karamanoglu Mehmetbey University, Karaman, Turkey; 3 Fruit and Vegetable Storage and Processing Department, The National Institute of Horticultural Research, Skierniewice, Poland

**Keywords:** Molecular biology, Plant sciences, Mathematics and computing

## Abstract

Biotic stress imposed by pathogens, including fungal, bacterial, and viral, can cause heavy damage leading to yield reduction in maize. Therefore, the identification of resistant genes paves the way to the development of disease-resistant cultivars and is essential for reliable production in maize. Identifying different gene expression patterns can deepen our perception of maize resistance to disease. This study includes machine learning and deep learning-based application for classifying genes expressed under normal and biotic stress in maize. Machine learning algorithms used are Naive Bayes (NB), K-Nearest Neighbor (KNN), Ensemble, Support Vector Machine (SVM), and Decision Tree (DT). A Bidirectional Long Short Term Memory (BiLSTM) based network with Recurrent Neural Network (RNN) architecture is proposed for gene classification with deep learning. To increase the performance of these algorithms, feature selection is made from the raw gene features through the Relief feature selection algorithm. The obtained finding indicated the efficacy of BiLSTM over other machine learning algorithms. Some top genes ((*S*)-*beta-macrocarpene synthase*, *zealexin A1 synthase*, *polyphenol oxidase I*, *chloroplastic*, *pathogenesis-related protein 10*, *CHY1*, *chitinase chem 5*, *barwin*, and uncharacterized *LOC100273479* were proved to be differentially upregulated under biotic stress condition.

## Introduction

Cereals constitute around half of the food production worldwide^1^. Maize (*Zea mays* L.), which originated in Mexico, is one of the most frequent cereals after wheat and rice worldwide^2^. It has supplied 1 × 10^9^ t of global food since 2013^3^. Maize, also called corn, play an essential role in supporting the increased human population through either direct consumption as dietary products or indirect use as livestock feed. Corn could also be a source of bioethanol production, acting as a renewable substitute for fossil fuel^4^.

Abiotic stresses, in conjunction with biotic stresses, may negatively affect maize production. Pathogens comprising fungal, bacterial, and viral are principally biotic stresses that cause heavy damage. The reduction in global maize yield due to biotic stresses is approximately 10%. During recent decades, the response of maize to abiotic and biotic stresses has been investigated through high-throughput omics approaches, especially transcriptomics. In this study, the dominant biotic stresses, threatening maize production, are further investigated in transcript levels by meta-analysis and artificial intelligence^2^.

Meta-analysis is a relatively inexpensive approach in which the results of independent studies are combined. The increased statistical power of meta-analysis and its generalizability may bypass the challenges associated with individual variations^5^. Meta-analysis in the bioinformatics field has been employed to determine differentially expressed genes (DEGs) under different treatments (e.g. normal and stress conditions) and uncover top genes producing fundamental molecules in confrontation with stress^6^.

A dramatic increase in microarray gene expression data, available via online resources, has been supported thanks to recent advances in high-throughput technologies. Therefore, combining multiple datasets can result in more reliable results. Thus, identifying DEGs under experimental conditions in plant breeding programs can be done using robust statistical methods. Microarray generates the expression of thousands of genes simultaneously with a minimal sample size^7^. One fundamental challenge of the microarray analysis is a high dimension result. Therefore, gene selection is necessary to remove redundant and irrelevant genes and reduce data dimensions that prevent over-fitted learning models^8^.

Artificial intelligence-based methods have started to be applied in many different areas to automatically perform tasks such as feature selection, feature extraction, classification, and prediction using existing data. Thanks to various artificial intelligence methods, learning from data is carried out. For this reason, mathematical transformations that modify the input data according to the output data are performed. With Machine learning, the sub-title of artificial intelligence, learning and decision-making can be performed by analyzing raw data. In the analysis process, the feature extraction task is essential regarding classification performance. Deep learning, another sub-title of Artificial Intelligence, is the developed version of Artificial Neural Networks, a Machine learning method based on neural networks. The main advantage of deep learning is that it automatically extracts high-level features without requiring a feature extraction step. While it is much more advantageous to use deep learning in applications with high data, machine learning algorithms can be used for less computational cost. Most of the deep learning-based classification applications use Convolutional Neural Networks (CNN) and need labelled data (i.e. supervised)^9, 10^.

Recently, artificial intelligence has also gained popularity in genomic studies. Clare and King^11^ presented a CNN-based architecture called DeepChrome to classify gene expression levels. In addition, the developed architecture could automatically learn combinatorial relationships between histone modification marks. The authors performed experimental studies on the REMC dataset containing 56 cell types. Qu et al.^12^ benefited from different feature extraction methods such as Auto-cross covariance, Khmer, and Parallel correlation pseudo amino acid composition to identify Pentatricopeptide repeat (PPR)-encoding genes and proteins that play a vital role in plant growth. Then they combined and mixed these features. They reduced the size of the blended features with the Max-Relevance-Max-Distance (MRMD) feature reduction technique. They classified the obtained reduced features with high accuracy with different machine learning methods (J48, Naïve Bayes (NB), and Random Forest (RF)).Chien et al.^13^ emphasized the importance of altering gene expression with T-DNA in rice functional gene studies. In this context, they developed an application to predict gene expression in T-DNA mutants using machine learning techniques. In the application developed in two layers, they coded nine features with PROMOTER and MIDDLE sequences in the first layer. They used these features with SVM. In the second layer, the minimum redundancy maximum relevance (mRMR) feature selection method and the LADTree algorithm were used to select and analyze the values selected in the first layer. This two-layer application, called TIMgo, showed 99.3% accuracy in its five-fold cross-validation experiment.

The present study was designed to perform gene selection by artificial intelligence, and its performance analysis. Classification of expressed genes under normal and biotic stress in maize using various feature selection algorithms was evaluated. Furthermore, the performance of the algorithms to deal with the data characterized as high-dimension and low sample-size data was surveyed. In the current study, two novel approaches to mining the gene expression of maize, comprising meta-analysis along with traditional machine learning and BiLSTM methods, were integrated for the first time to recognize the most informative genes in response to biotic stress.

## Materials and methods

### Data collection

Microarray gene expression profiles of the datasets related to biotic stress in maize (Table 1) were searched on the NCBI Gene Expression Omnibus database (GEO, https://www.ncbi.nlm.nih.gov/geo/). An Affymetrix platform GPL4302 was applied for data collection, and the search was restricted to the data in CEL format. Raw expression profiles were downloaded by the GEOquery package in R version 4.1.2.

**Table 1:** Microarray datasets from NCBI-GEO considered for meta-analysis and machine learning in mize subjected to biotic stresses.

Accession	Number of samples	Title of the experimental studies
GSE48406	21	Maize gene expression during infection with the *Ustilago maydis* mutant for cluster 19A and subdeletions for individual genes of cluster 19A (ID: 200048406)
GSE48536	9	Maize gene expression after infection of *Ustilago maydis* SG200 and SG200Δtin2 (ID: 200048536)
GSE40052	20	Expression data from Maize leaves post inoculation of *Sporisorium reilianum* (ID: 200040052)
GSE29747	6	*Sporisorium reilianum* Infection Changes Inflorescence and Branching Architectures of Maize (ID: 200029747)
GSE31188	12	Maize gene expression during infection with *Colletotrichum graminicola* (ID: 200031188)
GSE27626	12	Responses of *Zea mays* root tissue to inoculation with the necrotrophic root pathogen *Phytophthora cinnamomi* (ID: 200027626)
GSE19559	9	Maize gene expression during infection with *Ustilago maydis* strain SG200∆fox1 (ID: 200019559)
GSE19501	8	Maize gene expression during infection with *Fusarium moniliforme* (ID: 200019501)
GSE12892	6	Maize gene expression during infection with *Ustilago maydis* strain SG200Dpep1 (ID: 200012892)
GSE10023	39	Maize gene expression during infection with *Ustilago maydis* (ID: 200010023)

### Data preprocessing and merging

All raw gene expression files were quantile normalized and background corrected using Robust Multichip Average (RMA), which is an efficient way in the affy Bioconductor package^14^. Normalization was conducted to remove technical variations in experimental conditions. The combining of datasets from multiple experiments results in batch-specific bias variations that necessitate being eliminated. Thus, after preprocessing, the datasets were merged, and batch effect removal among different datasets was performed using the ComBat function in the SVA R package^15^, an empirical Bayes method. RMA and ComBat functions were performed using default parameters considered in the corresponding packages.

### Artificial intelligence-based gene recognition

Analyzing the data obtained by laboratory or different sensors, and accordingly, classification and prediction applications have been a research area for a long time. In such studies, instead of an expert's decision, the development of expert devices provides faster and easier solutions. In this sense, thanks to artificial intelligence, it is aimed for a machine to learn or solve a machine learning algorithm, e.g. Naive Bayes (NB), K-Nearest Neighbor (KNN), Ensemble, Support Vector Machine (SVM), and Decision Tree (DT), etc. have been extensively used. These algorithms provide classification and regression outputs by analyzing the input data. Machine learning is a rapidly evolving field with various applications in computer science. Deep learning, which is a branch of machine learning, has become easily applicable thanks to the developments in computer technology. In addition, deep learning-based data analysis applications provide superiority to traditional machine learning methods^16–18^. This study also compares deep learning and machine learning methods in terms of maize gene recognition performance. In this context, the recognition performances of BiLSTM, which is a modification of Deep Learning-based Recurrent Neural Networks (RNN) networks, and SVM, KNN, DT, NB, and Ensemble methods, which are traditional machine learning algorithms, will be evaluated. This section contains information about these artificial intelligence methods.

Machine learning and deep learning algorithms can be used in applications such as diagnosing plant diseases, determining irrigation and fertilization time, classification of genes, classification of different cultivars of fruits and vegetables in agriculture field.

The maize gene features used in this study have different upper and lower limits. Therefore, normalization for each feature may boost the learning success of artificial intelligence techniques^19^. For this reason, before the use of artificial intelligence techniques, the min–max normalization technique defined in Eq. (1) was used for the normalization of gene properties.

1 $${x}_{norm}=\frac{x-{x}_{min}}{{x}_{max}-{x}_{min}}$$

### Machine learning-based gene recognition

For the development of traditional machine learning methods applications for classification or regression purposes, the features of the data must have a strong discriminating ability. For this reason, the most robust distinguishing features should be selected by an expert. So feature selection and feature extraction is the most critical part of machine learning^20^. Depending on the data or application, these distinguishing features differ. In this gene recognition study, gene characteristics were selected according to SVM, KNN, DT, NB and Ensemble methods were used to classify these features. In this section, brief information about these traditional methods is shared.

SVM^21^, is a supervised learning method that shows high discrimination performance on small datasets by creating hyperplanes. SVM can efficiently perform nonlinear classification using a method called kernel trick. KNN^22^, performs classification according to the k nearest neighbors to the test data in the feature space. DT is a supervised learning method that uses graphs similar to a tree structure (nodes, branches, and leaves) to learn simple decision rules from data features. NB^23^, is a supervised learning algorithm that statistically distinguishes data according to the Bayesian method with the assumption of naive independence. Ensemble Learning^24^, combines the multiple learning models. That is, it improves machine learning results by blending the predictions of several machine learning-based algorithms^25–28^.

**Figure 1:**
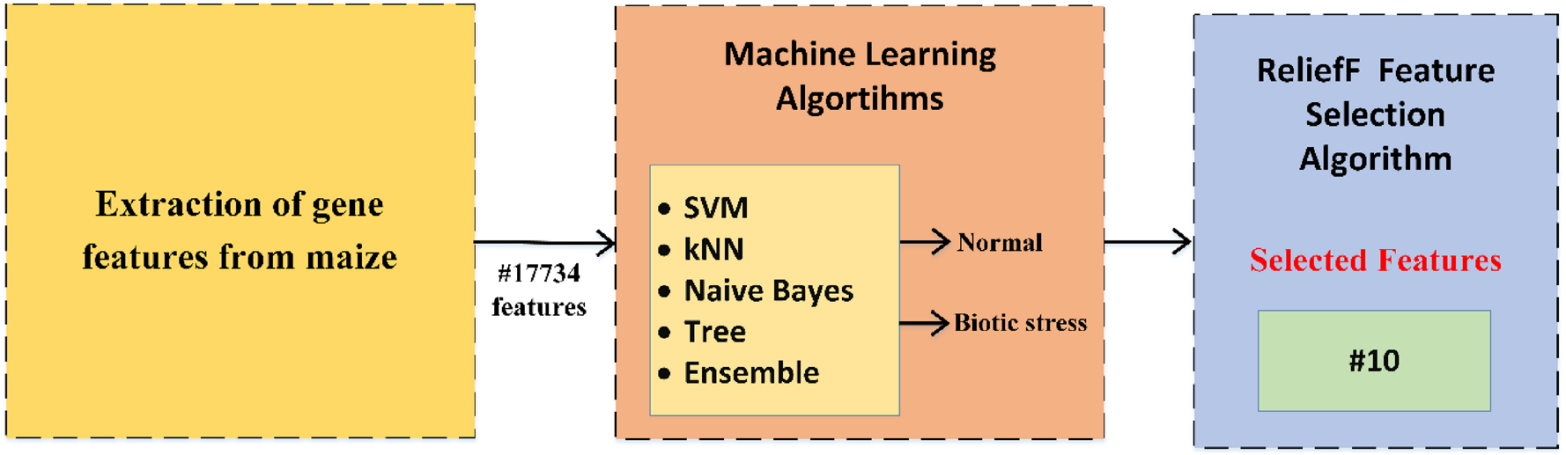
Block diagram of the proposed method for comparative artificial intelligence models in maize under biotic stress.

In the experimental study, a large number of genes (17,734) are present in the merged dataset, which is a matrix of gene expression values in maize under control and stress conditions. However, a large number of features often necessitate feature selection. Because raw data may contain irrelevant and unnecessary information. This negatively affects the learning success of the learning algorithm. Thanks to the feature selection algorithms, the size of the training samples is reduced. As a result, the computational burden and complexity of classification algorithms are reduced. Feature selection allows machine learning algorithms to focus on the most relevant features, therefore, feature selection is crucial for pattern recognition and machine learning applications^29, 30^. This study also gives selected features to machine learning methods. A Relief feature selection algorithm^29, 31^ is used for feature selection. The general representation of machine learning-based gene classification is shown in Fig. 1.

### Deep learning-based gene recognition: RNN-based BiLSTM network

In data-driven artificial intelligence algorithms, traditional methods such as ANN, SVM, and KNN have provided successful applications by modelling complex relationships between data. However, these methods do not consistently achieve global optimization. Especially with time-dependent data, traditional methods such as ANN are not capable. In this context, a Recurrent Neural Network (RNN), which is an advanced version of ANN, takes into account temporal dependence while extracting features from the data. In short, RNN is a kind of enhanced ANN that can convert time-related information into features^32, 33^. Although RNN is suitable for short-term dependent problems, it fails for long-term dependent problems^34^. RNN models suffer from the problem of loss of prior information over time when processing long interrelated sequences. Especially as the depth of the network increases, this problem prevents the network from learning. This problem in the training process is also well-known as the vanishing gradient problem. Hence, Long short-term memory (LSTM), a variation of RNN, was proposed byHochreiter and Schmidhuber^35^ to solve the vanishing gradient problem. LSTM performs better than RNN on long-time series data in the training process because it has a memory cell structure to store previous information.

The LSTM network first emerged with a memory cell, then Gers et al.^36^ developed LSTM by adding a forget gate. LSTM uses gates and memory cells so that the error gradient decreases over time. These cells ensure that the error value is preserved in the backpropagation step and previous information is remembered. In this way, previous information and existing data can be associated. The structure of LSTM has one memory cell ($c$) and three gates. These gates are the input gate ($i$), the forget gate ($f$), and the output gate ($o$). The memory cell consists of the previous memory (${c}_{t-1}$) and the newly modulated memory (${c}_{t}$). The input gate controls the values given as input to the LSTM and transmits them to the memory cell. The forget gate controls the memory cell to remove redundant and store information. The output gate controls the output of the current state^37, 38^. As a result of all these steps, the current hidden state ℎ*t* is calculated using the current input ${x}_{t}$ and the hidden state (${h}_{t-1}$) of the previous input (${x}_{t-1}$). The relationship between the input and output information of the LSTM and the behavior of the gates are expressed by the following equations:

2 $${i}_{t}={\sigma }_{g}({W}_{i}{x}_{t}+{U}_{i}{h}_{t-1}+{b}_{i})$$

3 $${f}_{t}={\sigma }_{g}({W}_{f}{x}_{t}+{U}_{f}{h}_{t-1}+{b}_{f})$$

4 $${o}_{t}={\sigma }_{g}({W}_{o}{x}_{t}+{U}_{o}{h}_{t-1}+{b}_{o})$$

5 $${c}_{t}={f}_{t}*{c}_{t-1}+{i}_{t}*{\sigma }_{c}({W}_{c}{x}_{t}+{U}_{c}{h}_{t-1}+{b}_{c})$$

6 $${h}_{t}={o}_{t}*{\sigma }_{h}({c}_{t})$$

**Figure 2:**
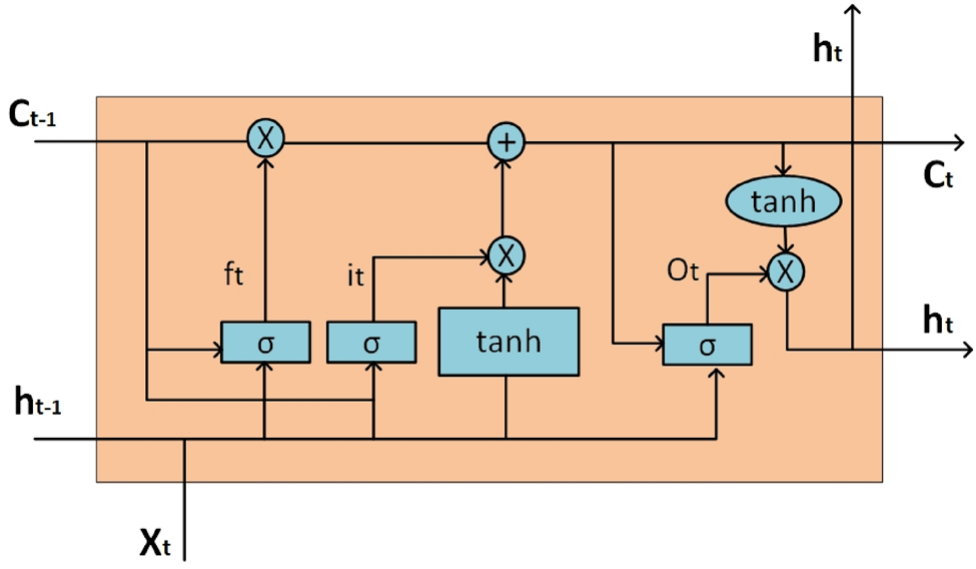
Structure of an LSTM network for comparative artificial intelligence models in maize under biotic stress.

In Eq. (2–6), $W$ denotes the weights of the hidden layer, $b$ the bias value, $\sigma (\dots )$ the sigmoid activation function, and $tanh(\dots )$ the hyperbolic tangent function. The $\otimes$ function displays multiplication by element. The structure of the architecture of the LSTM network is shown in Fig. 2. Unlike LSTM, Bidirectional LSTM (BiLSTM) considers the forward and backward data of the sequence to achieve higher prediction accuracy. So it is a combination of forward LSTM and backward LSTM. While LSTM uses only past information, BiLSTM is based on past and future information. Therefore, BiLSTM has a high generalization ability to provide a stronger prediction^39^.

### BiLSTM-based gene recognition application

**Figure 3:**
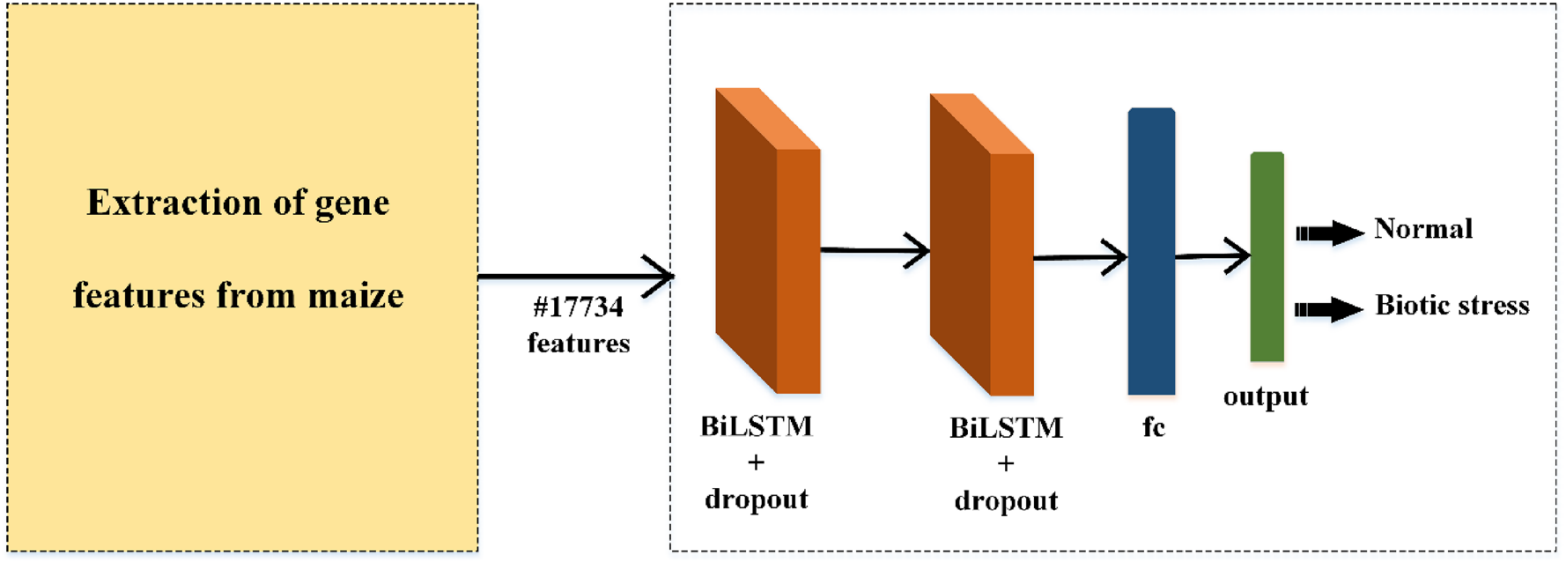
Proposed BiLSTM based gene recognition network for comparative artificial intelligence models in maize under biotic stress.

Although the LSTM and BiLSTM networks were designed to analyze temporal data, they also performed highly in non-temporal data^40–42^. In this context, this study also does not use temporal data but uses the powerful feature extraction capability of BiLSTM. The structure of the deep architecture designed to recognize maize genes is given in Fig. 3. The gene features are given to the BiLSTM network as input. As output, the system is trained through Fully Connected (FC) layers connected to the BiLSTM network, and Softmax layers determine the gene type.

In the RNN architecture shown in Fig. 3, two BiLSTM layers are used. A network that provides a deeper and more powerful classification is aimed, thanks to the cascading BiLSTM architecture. Both BiLSTM layers contain dropout. Dropout assigns a random value of 0 to input data during the training phase. The aim is to prevent overfitting. The extracted features are transmitted to the final layer via the FC layer. Features and target values are associated with the FC layer. In the last layer, softmax performs classification by interpreting the FC output as probabilistic. The network parameters used during the training of this network are presented in Table 2.

**Table 2:** Proposed network parameters and training parameters for discrimination of control and biotic stress conditions in maize based on gene expression values.

BiLSTM-1			BiLSTM-2			FC	
Number of hidden units	State activation function	Gate activation function	Number of hidden units	State activation function	Gate activation function	Output mode	State activation function
200	$tanh$	$sigmoid$	200	$tanh$	$sigmoid$	last	$tanh$

Training parameters							
Optimizer	Gradient decay factor (${\beta }_{1}$)	Squared gradient decay factor (${\beta }_{2}$)	Max. epoch	Mini batch size	Initial learning rate ($\alpha$)	Epsilon ($\epsilon$)	
Adam	0.9	0.999	250	48	0.001	${10}^{-8}$	

The parameter values given in Table 2 were determined by trial and error. In the training phase, the network weights are optimized with the Adam Optimization algorithm^43^, an adaptive learning rate algorithm. Adam Optimizer provides superiority to other optimization algorithms with its relatively low memory requirement. The parameter update equation in the Adam Optimization algorithm is shown in Eqs. (7–9).

7 $${m}_{l}={B}_{1}{m}_{l-1}+\left(1-{\beta }_{1}\right)\nabla E({\theta }_{l})$$

8 $${v}_{l}={B}_{2}{v}_{l-1}+\left(1-{\beta }_{2}\right){[\nabla E\left({\theta }_{l}\right)]}^{2}$$

9 $${\theta }_{l+1}={\theta }_{l}-\frac{{\alpha m}_{l}}{\sqrt{{v}_{l}}+\epsilon}$$

$m$: Gradient moving averages, $v$: Squared gradient moving averages, $\theta$ : Network parameter to be updated, $\nabla E(\theta$): Gradient of the loss function, ${\beta }_{1}$: Gradient Decay Factor, ${\beta }_{2}$: Squared Gradient Decay Factor $\epsilon$: Epsilon, $\alpha$: Learning Rate, $l$ : Iteration Number.

## Results

In this work, we investigated the gene expression profiles of maize samples in control and biotic stress via several artificial Intelligence algorithms. The entire procedure is presented in Figs. 1 and 3. The metrics used to evaluate the performance of artificial intelligence algorithms are given between Eqs. (10–15^44^). Detailed results are presented in the sub-headings.

10 $$Accuracy=\frac{tp+tn}{tp+fp+tn+fn}x100$$

11 $$Precision=\frac{tp}{tp+fp}$$

12 $$Sensitivity=\frac{tp}{tp+fn}$$

13 $$Specificity=\frac{tn}{tn+fp}$$

14 $$F1-score=\frac{2tp}{2tp+fp+fn}$$

15 $$MCC=\frac{\left(tp*tn\right)-(fn*fp)}{\sqrt{\left(tp+fn\right)*\left(tn+fp\right)*\left(tp+fp\right)*(tn+fn)}}$$

$tp$: True Positive, $tn$: True Negative, $fp$: False Positive, $fn$: False Negative.

### Machine learning-based gene recognition

In machine learning algorithms, 80% of the data set was used for training and the remaining 20% for the test phase. To select appropriate and high-performance machine learning algorithms, the classification of the maize samples according to their gene expression under control and biotic stress was explored (Table 3). The classification was based on 17,734 genes corresponding to the GPL4032 platform. Gene expression profiles are qualitative types of data.

**Table 3:** Performance metrics of machine learning algorithms and BiLSTM model for discrimination of control and biotic stress conditions in maize based on gene expression values.

Models	Accuracy (%)	Sensitivity	Specificity	Precision	F1-Score	MCC
SVM	78.57	0.8235	0.7273	0.8235	0.8235	0.5508
KNN	82.14	0.8824	0.7273	0.8333	0.8571	0.6214
Naive Bayes	75.00	0.7647	0.7273	0.8125	0.7879	0.4855
Tree	75.00	0.8235	0.6364	0.7778	0.8000	0.4688
Ensemble	85.71	0.8235	0.9091	0.9333	0.8750	0.7174
BiLSTM	92.86	0.9412	0.9091	0.9412	0.9412	0.8503

Among the investigated algorithms, while Naive Bayes and Tree performed the worst with an average accuracy of 75%, Ensemble displays the highest accuracy with an average of 85.71% (Table 3). According to the results, the lowest performance in terms of sensitivity corresponded to the Naïve Bayes algorithm correctly detecting 0.7647 positive samples. Further, KNN has the highest Sensitivity criteria, with an average of 88.24%, respectively (Table 3).

**Figure 4:**
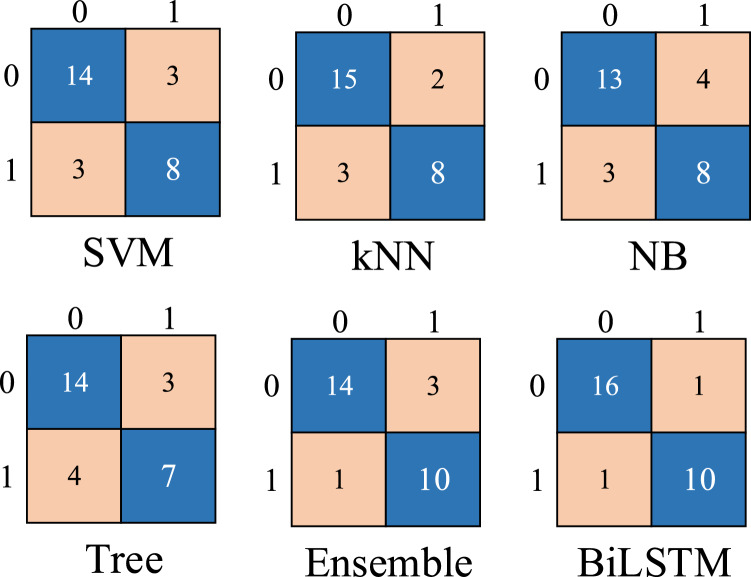
Confusion matrices of machine learning and deep learning models performed on gene expression profiles under biotic stress in maize.

The Tree algorithm turned out to be the weakest developed machine for the detection of the negative sample with 63.64% specificity. In contrast, the highest average specificity of 90.91% was obtained by Ensemble, followed by SVM, KNN, and Naive Bayes algorithms. Among these algorithms, Ensemble brought about the highest average precision, with an average of 93.33%. The Tree algorithm performed the weakest in terms of precision, equal to 77.78% (Table 3). Finally, according to the MCC and the F1 Score criteria, the Ensemble algorithm has the best performance (Table 3). Interestingly, while Tree had a relatively high performance of MCC criteria (80.0%), its F1 score was the lowest equal to 46.88%. The confusion matrices of machine learning algorithms are shown in Fig. 4. In the confusion matrix, the controlled maize gene was labelled 0 and the biotic stressed maize gene was labelled 1.

### Deep learning-based gene recognition: RNN-based BiLSTM network

**Figure 5:**
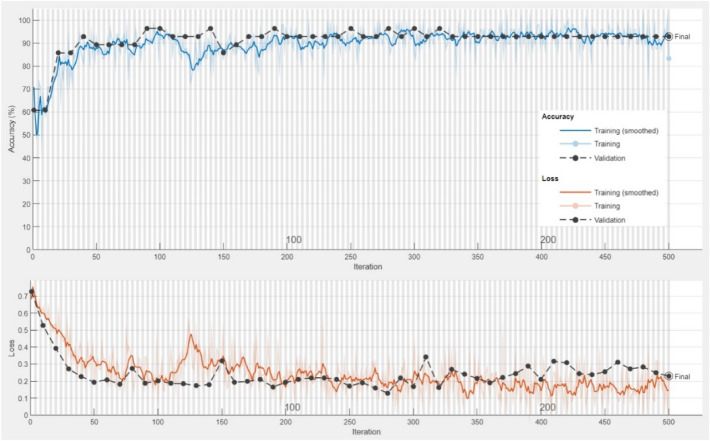
Training and loss graphics of BiLSTM model based on whole gene expression data (All genes (#17734)) in maize under biotic stress conditions.

Construction of the models was based on 80% of the dataset for BiLSTM and the resting 20% for testing the BiLSTM models. The number of hidden units of the BiLSTM classifier was set to 200. Training of the BiLSTM network was attained with Adam Optimizer. As well, the epoch numbers and batch size were set to 48 and 250, respectively. The initial learning rate of the BiLSTM network was set to 0.001 (Table 2). Figure 4 clarifies the confusion matrix of the BiLSTM model. Moreover, the results of BiLSTM on gene expression data are shown in Table 3. The average accuracy for BiLSTM classifiers was obtained to be 92.86%. As well, sensitivity, precision, and F1-score reached a high performance of 94.12%. Specificity and MCC parameters were slightly lower, with 90.91 and 85.03%, respectively (Table 3). As the learning process continues the accuracy of the generated training set increases (Fig. 5). As iteration progressed, the rising trend of accuracy slowed down, and the final accuracy was sustained at around 92%, reaching the highest accuracy of 92.86%. The training and validation curves showed a considerable overlapping confirming the acceptable performance of the model. In Fig. 5, with progress in iteration, the minimum Loss value in the training set reached 0.1, and the minimum in the case of the validation set was nearly in the range of 0.2 to 0.3.

**Figure 6:**
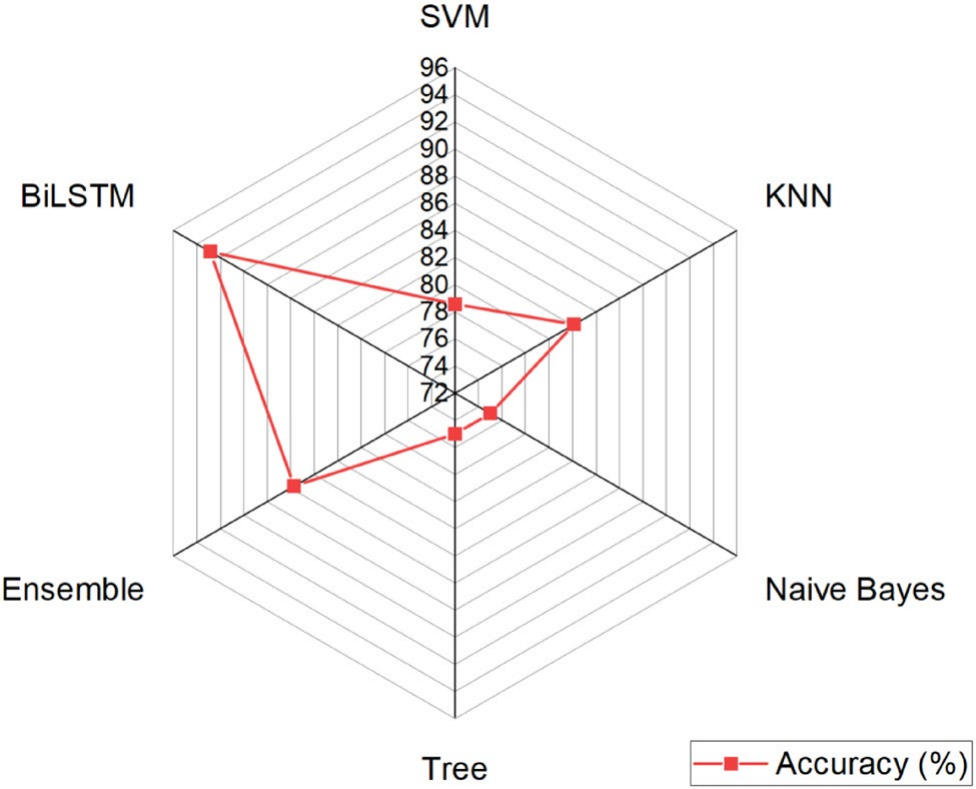
Classification accuracy of machine learning and deep learning models built based on gene expression values under biotic stress in maize.

The radar graph of the classification accuracy of machine learning and deep learning models was given in Fig. 6. The results showed that the model has satisfactory performance classification of maize samples according to their gene expression under control and biotic stresses.

### Analysis of top differentially expressed genes

Among the 17,734 genes in our data set, the 10 most effective genes in the classification of controlled and biotic stressed genes were determined by the Relief feature selection algorithm. We compared the 10 top genes identified by the Relief feature selection algorithm and the list of the genes that were differentially expressed (Table 4). Eight out of 10 genes were turned out to be upregulated, and two of them downregulated. The top genes were submitted to the DAVID tool (https://david.ncifcrf.gov/)^45, 46^ to determine the biological processes and pathways controlled by each gene.

**Table 4:** Expression of 10 top selected genes based on the relief feature selection algorithm in maize subjected to biotic stresses.

probe ID	Gene symbol	Gene description	logFC	adj.P.Val
Zm.14496.1.A1_at	LOC103641097	(S)-beta-macrocarpene synthase	4.427	2.05E−16
Zm.14226.1.A1_at	LOC100273457	zealexin A1 synthase	2.954	5.94E−15
Zm.3129.1.A1_at	LOC103641120	polyphenol oxidase I, chloroplastic	1.828	2.00E−14
Zm.10175.1.A1_at	LOC100280981	pathogenesis-related protein 10	2.837	3.31E−15
Zm.9486.1.A1_at	LOC100283469	CHY1	2.653	1.09E−14
Zm.1085.1.A1_a_at	LOC542525	chitinase chem 5	2.383	6.12E−13
Zm.2227.1.A1_at	LOC103652814	barwin	3.129	3.74E−12
Zm.2031.4.A1_at	LOC100282059	uncharacterized LOC100282059	− 0.729	2.47E−04
Zm.10090.1.A1_at	LOC100286331	senescence-associated protein DIN1	− 0.138	2.96E−05
Zm.11214.1.S1_at	LOC100273479	uncharacterized LOC100273479	1.008	7.34E−11

The gene *LOC103641097* was differentially expressed in control and biotic stress conditions in maize. This gene is involved in different biological processes, including diterpenoid biosynthetic process, terpenoid biosynthetic process, defence response to fungus, and KEGG pathways associated with Sesquiterpenoid and triterpenoid biosynthesis.

The next upregulated gene, *LOC100283469*, may contribute to the valine catabolic process and several KEGG pathways including valine, leucine, and isoleucine degradation, beta-Alanine metabolism, propanoate metabolism, carbon metabolism, and metabolic pathways.

Gene *LOC103652814* may play a role in the defense response to bacterium and fungi. Overexpression of *LOC542525* under biotic stress results in carbohydrate metabolic process and pathways related to metabolic pathways, and amino sugar and nucleotide sugar metabolism.

Gene *LOC100280981*, encoding a pathogenesis-related protein, contributes to the various biological process in defence response, abscisic acid-activated signalling pathway, and regulation of protein serine/threonine phosphatase activity.

The next upregulated gene *LOC103641120*, which is connected to isoquinoline alkaloid biosynthesis, tyrosine metabolism, biosynthesis of secondary metabolites pathways, and metabolic pathways.

*LOC100273479* was identified to be involved in oxidative stress and the hydrogen peroxide catabolic process. This gene has a molecular function connected to peroxidase activity, metal ion binding, and heme binding, and plays a role in metabolic pathways, phenylpropanoid biosynthesis, and biosynthesis of secondary metabolites. The following upregulated gene *LOC100273457* (*zealexin A1 synthase*) turned out to be involved in the defense response and diterpene phytoalexin biosynthetic process.

Gene *LOC100286331*, downregulated during biotic stress in maize, functionally was connected to catalytic activity. No biological processes and pathways were identified in the case of the uncharacterized downregulated gene *LOC100282059*.

## Discussion

This study investigated the efficacy of machine learning and the BiLSTM network to construct an optimal classifier that can classify maize samples at the transcriptomic level in control and biotic stress condition. Moreover, 10 top genes were selected based on the Relief feature selection algorithm, listed in Table 4, to be further investigated in detail. Overexpression and down expression of the selected genes were determined from the list of differentially expressed genes (DEGs) performed using the limma package. The result summarized the down and up regulated genes as 7744 and 4167, respectively.

Gene expression datasets are High Dimension and Low Sample Size (HDLSS) and less research has been done on machine learning models in this field. Hence, Deep Learning is being investigated on a multi-omics dataset^7^. In this study, gene expression metrics of 14 datasets were merged using meta-analysis. Then, several algorithms of machine learning and BiLSTM were applied to classify maize samples based on the gene expression data. It was concluded that BiLSTM performed better than the machines which were trained.

Machine learning has recently been used in bioinformatics research. The Machine Learning algorithms can be divided into Statistical Learning (Naïve Bayes), perceptron-based algorithms (Multi-layered Perceptron, Neural Network), and logic-based algorithms (e.g. Random Forest, Decision Trees)^47^. The classification models discussed in this research include SVM, KNN, Naïve Bayes, Tree, Ensemble, and BiLSTM.

The binary classification base on SVM depends on forming a hyperplane employing the kernel function. A paramount preference for adopting SVM is tackling the outliers^48^. The KNN algorithm assumes that similar things within a dataset exist nearby. Although perception and implementation of the KNN algorithm are easy, the fundamental principle in choosing the value of k is a shortcoming. As well, it is sensitive to the function of similarity or distance. A Decision Tree is a decision support tool of nodes and branches. Though it is mainly applied due to its effectiveness and calculation speed, this algorithm is highly disposed to underfitting and overfitting to fit the model^49^. A random forest consists of a group (an ensemble) of individual decision trees. The Random Forest is one of the most powerful machine learning algorithms that can be used for classification. Naïve Bayes is the statistical classification model established based on the assumption of independence of all features in the dataset.

Generally, neural networks and SVM perform better for continuous and multi-dimensional features. On the other hand, the logic-based algorithms e.g., the rule learners and decision trees, have better performance for categorical or discrete features. One way to identify the algorithm's outperformance over the other is through the validation process and estimating the model's accuracy. Accuracy was considered as the notable criterion to evaluate the quality of predictive models measuring the ratio of evaluated correct predictions to the total number of cases. While the accuracy obtained through the BiLSTM model was 92.86%, Ensemble was the most accurate model for the classification of maize samples based on gene expression, having high accuracy of 85.71% in control and biotic stress conditions. Moreover, sensitivity, specificity, precision, F1-Score, and MCC were also implemented to evaluate the models.

The gene *TPS11* (*LOC103641097*), encoding bicyclic sesquiterpene (S)-beta-macrocarpene, has been reported to contribute to the regulation of plant defence. Köllner et al.^50^ indicated the expression of *TPS11* in the root of the maize resulted in the enzyme production of (*S*)-β-macrocarpene and (*S*)-β-bisabolene. It has been reported that the expression of *terpene synthases TPS10*, *TPS11*, and *TPS21* may support the terpenoid production and serve as resistance to insect attacks^51^. Therefore, the expression pattern of such a gene can be a potential distinctive biomarker for identifying the tolerant genotypes against biotic stress in maize.

The role of *LOC100283469* in abiotic stress-tolerant has previously been reported. Dong et al.^52^ reported that *CHY1* mutants showed reduced cold tolerance in Arabidopsis. Expression of CHY1 can lead to hydrolysis of HIBYL–CoA to CoA and b-hydroxyisobutyrate throughout Val catabolism^53^. Wang et al.^54^ confirmed that significant enrichment in pantothenate and CoA biosynthesis, besides the valine, leucine, and isoleucine biosynthesis pathways, can be significantly induced by salt stress. To the best of our knowledge, here is the first report of CHY1 as the regulatory gene in biotic stress conditions.

Gene LOC103652814 encodes BARWIN, which is a conserved domain of PR4 protein and its function in plant response to biotic and abiotic stress, has been identified. PR4 protein expressions are induced by *Magnaporthe grisea* infection in rice. As well, this gene was observed to be responsive in the presence of at least one of the abiotic stress, including drought, salt, cold, wounding, heat shock, and ultraviolet^55^.

The gene *LOC542525* encodes chitinase chem 5, which may be involved in several growth processes and stress responses. Chitin is a component of the fungal cell wall; however, plants do not produce chitin. Hence, it has been indicated that plant chitinases hydrolyze the cell walls of pathogens and release elicitors, pathogen signal metabolites recognized by plant cells, which may trigger plant defences^56^. Infection of the plant tissue with fungal pathogens can induce the chitinase expression in many plants^56^. It was indicated that overexpression of the chitinase could enhance protection against fungal infection in transgenic tobacco^57^. López and Gómez-Gómez^58^ demonstrated the promotion of the transcription of some *chitinase* genes under various abiotic stresses, including drought, cold, and salt^58^.

Expression of gene *LOC100280981* upon either the attacks of pathogens or environmental stress identified the involvement of Pathogenesis-related protein in plant defence mechanisms^59^.

*LOC103641120* (PPO) is a ubiquitous metalloproteinase in plants with a mediating act in plant resistance against biotic and abiotic stresses^60^. To understand the expression of PPO in tomato under water stress, two members out of the seven-member PPO gene family were found upregulated in abscission zones of leaf petioles^61^.

Our results were in line with Mantri et al.^62^, who determined the transcriptional profile of DEGs in chickpea in response to drought, cold, and high-salinity stress. They reported the repression of *LOC100286331* (DY396338) in roots, leaves, and flowers of the tolerant genotype, while DY396338 was upregulated in the roots of the susceptible genotype. Downregulation of *LOC100286331* upon biotic stress in maize may explain that plants avoid death through a tolerance mechanism.

Gene *LOC100273457* produces zealexin A1 synthase, which is involved in the production of the antifungal phytoalexin zealexin A1. This enzyme oxidizes (S)-beta-macrocarpene through intermediates of alcohol and aldehyde to form zealexin A1^63^. Shen et al.^64^ demonstrated the role of *LOC100273457* in increased chemical defence while occurring the blast fungus in transgenic rice. They noted *LOC100273457* might produce 15,16-epoxy-syn-pimaradien-19-ol to inhibit the germination of spores and the formation of appressorium of *Magnaporthe oryzae* in rice^64^.

## Conclusions

In this contribution, an integrated meta-analysis and machine learning approaches, as well as deep learning, were adopted to classify maize samples based on the gene expression profiles under control and biotic stress. Furthermore, gene ontology and KEGG pathways of top 10 genes, selected based on the Relief feature selection algorithm, were performed to determine underlying mechanisms in biotic stress conditions. As a result, the superiority of BiLSTM over other machine learning algorithms was demonstrated. Moreover, the results of gene selection indicated several potential resistance genes, among which (S)-beta-macrocarpene synthase, zealexin A1 synthase, polyphenol oxidase I, chloroplastic, pathogenesis-related protein 10, CHY1, chitinase chem 5, barwin, and uncharacterized LOC100273479 are more important than others.

## Data Availability

The datasets analyzed during the current study are available in the GSE48406, GSE48536, GSE40052, GSE29747, GSE31188, GSE27626, GSE19559, GSE19501, GSE12892, and GSE10023, repository, https://www.ncbi.nlm.nih.gov/geo/.
